# Survival Following CDK4/6 Inhibitor Therapy for Hormone Receptor–Positive, *ERBB2*–Negative Metastatic Breast Cancer

**DOI:** 10.1001/jamanetworkopen.2024.61067

**Published:** 2025-02-21

**Authors:** Pier Paolo Maria Berton Giachetti, Stefania Morganti, Sara Gandini, Fabiola Giudici, Antonio Marra, Eleonora Nicolò, Emma Zattarin, Chiara Corti, Laura Boldrini, Annarita Verrazzo, Caterina Sposetti, Maria Grazia Razeti, Ambra Carnevale Schianca, Roberta Scafetta, Beatrice Taurelli Salimbeni, Angela Esposito, Paola Zagami, Dario Trapani, Bianca Malagutti, Roberta Caputo, Claudio Vernieri, Elisabetta Munzone, Simone Scagnoli, Andrea Botticelli, Matteo Lambertini, Mario Giuliano, Michelino De Laurentiis, Giulia Viale, Giampaolo Bianchini, Giuseppe Curigliano, Carmine De Angelis, Carmen Criscitiello

**Affiliations:** 1 Early Drug Development for Innovative Therapies, European Institute of Oncology IRCCS, Milan, Italy; 2 Department of Oncology and Haemato-Oncology, University of Milano, Milan, Italy; 3 Department of Medical Oncology, Dana-Farber Cancer Institute, Boston, Massachusetts; 4 Harvard Medical School, Boston, Massachusetts; 5 Broad Institute of MIT and Harvard, Cambridge, Massachusetts; 6 Department of Experimental Oncology, IEO, European Institute of Oncology IRCCS, Milan, Italy; 7 Cancer Epidemiology Unit, Centro di Riferimento Oncologico di Aviano IRCCS, Aviano, Italy; 8 Department of Medicine, Division of Hematology-Oncology, Weill Cornell Medicine, New York, New York; 9 Department of Oncology and Hematology, Azienda Ospedaliero-Universitaria di Modena, Modena, Italy; 10 Medical Oncology Department, Fondazione IRCCS Istituto Nazionale dei Tumori, Milan, Italy; 11 Department of Breast and Thoracic Oncology, Division of Breast Medical Oncology, Istituto di Ricovero e Cura a Carattere Scientifico Pascale, Naples, Italy; 12 Department of Internal Medicine and Medical Sciences, School of Medicine, University of Genova, Genova, Italy; 13 Department of Medical Oncology, UOC Clinica di Oncologia Medica, IRCCS Ospedale Policlinico San Martino, Genova, Italy; 14 Medical Oncology, Fondazione Policlinico Universitario Campus Bio-Medico, Via Alvaro del Portillo 200, 00128, Roma, Italy; 15 IFOM ETS, the AIRC Institute of Molecular Oncology, Milan, Italy; 16 Division of Medical Senology, Research Unit in Medical Senology, European Institute of Oncology IRCCS, Milan, Italy; 17 Department of Radiological, Oncological and Pathological Science, Sapienza University of Rome, Rome, Italy; 18 Department of Clinical Medicine and Surgery, University Federico II, Naples, Italy; 19 Università Vita-Salute San Raffaele, Milan, Italy; 20 Department of Medical Oncology, IRCCS Ospedale San Raffaele, Milan, Italy

## Abstract

**Importance:**

Endocrine therapy (ET) combined with cyclin-dependent kinase 4/6 inhibitor (CDK4/6i) agents is the standard first-line treatment for patients with hormone receptor–positive, *ERBB2* (formerly *HER2* or *HER2/neu*)–negative metastatic breast cancer. However, optimal therapy after tumor progression to ET plus CDK4/6i remains unclear.

**Objective:**

To evaluate progression-free survival (PFS) and overall survival (OS) in the clinical practice setting in patients with hormone receptor–positive, *ERBB2*–negative metastatic breast cancer following progression with ET plus CDK4/6i.

**Design, Setting, and Participants:**

The multicenter retrospective cohort study included 506 patients diagnosed with hormone receptor–positive, *ERBB2*–negative metastatic breast cancer between April 22, 2015, and January 31, 2023, and who received ET-based or chemotherapy (CT)-based treatment following progression during ET plus CDK4/6i. Outcomes were analyzed based on treatment type, clinicopathologic features, and the duration of prior CDK4/6i therapy.

**Main Outcomes and Measures:**

The primary end point was PFS in the clinical practice setting, defined as the time between the initiation of the first systemic treatment on tumor progression to ET plus CDK4/6i treatment and the detection of disease progression or patient death from any cause. The secondary end point was OS in the clinical practice setting, defined as the time interval between tumor progression during ET plus CDK4/6i treatment and patient death from any cause.

**Results:**

In 506 women (median age at diagnosis, 52.4 [IQR, 44.6-62.8] years) diagnosed with hormone receptor-positive, *ERBB2*–negative metastatic breast cancer progressing during ET plus CDK4/6i, independent factors associated with poorer PFS outcomes were visceral metastases (hazard ratio [HR], 1.45; 95% CI, 1.17-1.80; *P* = .008) and de novo metastatic disease (HR, 1.25; 95% CI, 1.01-1.54; *P* = .04). A longer duration of CDK4/6i therapy (OS HR, 0.55; 95% CI, 0.41-0.73; *P* < .001) and an older age (PFS HR, 0.99; 95% CI 0.98-1.00; *P* = .03) were associated with better outcomes. Compared with oral CT, both intravenous CT- and ET-based treatments were associated with shorter PFS (intravenous CT: hazard ratio [HR], 1.45; 95% CI, 1.11-1.89; *P* = .006; everolimus plus exemestane: HR, 1.38; 95% CI, 1.06-1.78; *P* = .02; ET only: HR, 1.38; 95% CI, 1.05-1.89; *P* = .02). A duration of CDK4/6i treatment exceeding 12 months was associated with longer OS (HR, 0.55; 95% CI, 0.41-0.73; *P* < .001). Among patients with visceral metastases, intravenous CT was associated with shorter OS compared with oral CT (HR, 1.52; 95% CI, 1.03-2.24; *P* = .04).

**Conclusions and Relevance:**

In this cohort study, the duration of tumor control achieved with CDK4/6i-based therapy and the presence of visceral metastases emerged as key factors that may affect treatment decision. Oral CT may offer potential benefits for specific patient subgroups.

## Key Points

**Question:**

Can post–cyclin-dependent kinase 4/6 inhibitor (CDK4/6i) therapy be optimized based on clinicopathologic characteristics in patients with hormone receptor–positive, *ERBB2* (formerly *HER2* or *HER2/neu*)–negative metastatic breast cancer?

**Findings:**

In a cohort study of 506 patients with hormone receptor–positive, *ERBB2*–negative metastatic breast cancer that progressed during endocrine therapy and CDK4/6i agents, younger age, de novo metastatic disease, and visceral involvement were independent factors associated with shorter progression-free survival. Additionally, a duration of CDK4/6i treatment exceeding 12 months was associated with significantly longer overall survival.

**Meaning:**

The findings of this study suggest that duration of prior CDK4/6i therapy and the presence of visceral involvement are key factors associated with hormone receptor–positive, *ERBB2*–negative metastatic breast cancer in patients progressing while receiving endocrine therapy plus CDK4/6i.

## Teaser

This cohort study examines the association of treatment strategy and clinicopathologic factors with progression-free and overall survival in patients with hormone receptor–positive, ERBB2-negative breast cancer with metastasis after treatment with endocrine and cyclin-dependent kinase 4/6 inhibitor therapy.

## Introduction

Breast cancer remains a leading cause of cancer morbidity and mortality, with hormone receptor–positive, *ERBB2*–negative (formerly *HER2* or *HER2/neu*) metastatic breast cancer being the most common subtype.^1,2,3,4^ Cyclin-dependent kinase 4/6 inhibitor inhibitors (CDK4/6i) combined with endocrine therapy (ET) have established efficacy as first-line treatment for hormone receptor–positive/*ERBB2*–negative metastatic breast cancer,^5^ and their introduction results in improved progression-free survival (PFS) and overall survival (OS) compared with ET only.^6,7,8,9,10,11,12,13,14^

Despite these advancements, most patients with hormone receptor–positive/*ERBB2*–negative metastatic breast cancer experience disease progression during first-line ET plus CDK4/6i treatment due to the development of acquired tumor resistance. In this setting, the optimal therapeutic strategy following progression during ET plus CDK4/6i treatment remains unclear, with limited data available to guide subsequent treatment choices.^15^ Current guidelines recommend assessing for actionable mutations (eg, *PIK3CA*, *ESR1*, *AKT*, *PTEN*) and conducting germline testing for *BRCA1/2* or *PALB2* mutations on tumor progression during ET plus CDK4/6i therapy.^16,17^ Therapeutic options include ET with or without targeted agents (eg, alpelisib, capivasertib, everolimus), PARP inhibitors for patients with germline *BRCA1/2* mutations, and chemotherapy (CT). In cases of extensive visceral involvement or poor response to first-line therapy, antibody-drug conjugates, such as trastuzumab deruxtecan, as found in the DESTINY Breast-06 trial, may be considered.^16,18,19,20,21,22,23,24,25,26,27,28,29^

Given the variety of available therapies, data from the clinical practice setting are crucial for informing clinical decisions after tumor progression during ET plus CDK4/6i therapy. Herein, we present the results of a multicenter retrospective cohort study aimed at identifying clinicopathologic factors potentially associated with survival outcomes and optimizing postprogression treatment strategies in patients with hormone receptor–positive/*ERBB2*–negative metastatic breast cancer.

## Methods

### Study Design and Participants

This multicenter retrospective cohort study included patients with hormone receptor–positive/*ERBB2*–negative metastatic breast cancer that progressed during first-line or second-line ET plus CDK4/6i therapy. Patients treated in the first-line setting could have endocrine-sensitive (aromatase inhibitors) or endocrine-resistant (fulvestrant) disease. Eligible patients had hormone receptor–positive (ER≥1% and/or PR≥1%), *ERBB2*-negative (defined in this study as immunohistochemistry score of 0, 1+, or 2+ without *ERBB2* gene amplification at fluorescence in situ hybridization on the primary tumor or metastasis biopsy, when available) metastatic breast cancer, with a documented date of metastatic relapse. Inclusion criteria required at least one systemic treatment after progression during ET plus CDK4/6i treatment. Patients with *ERBB2*–positive disease, those receiving subsequent CDK4/6i treatment after progression, or those who received therapies other than CT-based or ET-based options (eg, antibody-drug conjugates, immunotherapy, poly [ADP-ribose] polymerase inhibitor) were excluded. Additionally, patients starting a new treatment due to toxic effects rather than because of tumor progression during ET plus CDK4/6i therapy were excluded. Data were collected from medical records for consecutive patients diagnosed with metastatic breast cancer between April 22, 2015, and January31, 2023. All patients provided informed consent. The study was approved by the institutional review boards of all participating centers. This study followed the Strengthening the Reporting of Observational Studies in Epidemiology (STROBE) reporting guideline for cohort studies.^30^

### End Points

The primary end point was progression-free survival (PFS) in the clinical practice setting, defined as the time between the initiation of the first systemic treatment during tumor progression to ET plus CDK4/6i treatment and the detection of disease progression or patient death from any cause.^31^ The secondary end point was overall survival (OS) in the clinical practice setting, defined as the time interval between tumor progression during ET plus CDK4/6i treatment and patient death from any cause.^31^ Exploratory analyses included PFS and OS according to visceral involvement. Data cutoff for analysis was April 11, 2024.

### Participating Institutions

Six Italian referral institutions participated in the SISTER project. The institutions were European Institute of Oncology IRCCS, Milan; Istituto Nazionale dei Tumori IRCCS, Milan; Istituto Nazionale Tumori Fondazione Pascale IRCCS, Naples; Policlinico Umberto I IRCCS, Rome; IRCCS Policlinico San Martino Hospital, Genoa; and Istituto San Raffaele IRCCS, Milan.

### Statistical Analysis

Descriptive statistics were used to summarize patient demographic characteristics, disease characteristics, and treatment variables. Continuous variables are reported as medians (IQRs), and categorical variables are presented as frequencies and percentages. Associations of categorical variables were assessed using the Pearson χ^2^ test or Fisher exact test, and associations of continuous and categorical variables by using the Wilcoxon-Mann-Whitney test. Survival was estimated with the Kaplan-Meier method, with log-rank tests for comparisons. Cox proportional hazards regression models were used for univariate and multivariable analyses to estimate hazard ratios (HRs) and 95% CIs, incorporating prespecified established variables, such as age, metastatic disease status (de novo vs relapsed), CDK4/6i treatment duration, treatment type after ET plus CDK4/6i, or visceral metastases. Proportional hazards assumptions were tested using Schoenfeld residuals, with no violations detected.^32^ A significance threshold of *P* < .05 (2-tailed, unpaired) was applied. Statistical analyses were conducted using R, version 4.2.3 (R Foundation for Statistical Computing).

## Results

### Study Population and Subsequent Therapies After CDK4/6i

A total of 506 women (median age at diagnosis, 52.4 [IQR, 44.6-62.8] years) were included in this study, of whom 342 (67.6%) received ET plus CDK4/6i agents as the first line of treatment and 164 (32.4%) received ET plus CDK4/6i agents as the second line of treatment. Patients’ baseline characteristics are reported in Table 1. More than one-quarter of the patients (26.9%) were diagnosed with de novo metastatic disease, with carcinoma of no special type/invasive ductal carcinoma being the most prevalent breast cancer histologic subtype (73.7%). Patients who received ET plus CDK4/6i agents as first-line treatment were younger (60.6 vs 63.0 years; *P* = .009) and displayed a better Eastern Cooperative Oncology Group (ECOG) performance status (ECOG 0: 84.5% vs 72.6%; *P* = .01) compared with those who received CDK4/6i agents as second-line treatment. The median duration of treatment with ET plus CDK4/6i agents was 12.2 (IQR, 5.9-21.1) months, with a longer duration observed in patients who received CDK4/6i as first-line (13.4; IQR, 7.0-21.3 months) compared with second-line (8.8 months; IQR, 4.4-20.4 months) treatment (*P* = .006). Most patients (67.2%) received palbociclib, followed by ribociclib (23.1%) and abemaciclib (9.7%). Most of the patients (78.5%) were postmenopausal when ET plus CDK4/6i was started. All premenopausal patients received a luteinizing hormone-releasing hormone agonist in addition to ET plus CDK4/6i.

**Table 1.** Clinical Features of 506 Women According to Their CDK4/6i Treatment Lines

Characteristic	Total, No. (%)	CDK4/6i therapy, No. (%)		*P* value
		First line (n = 342 [67.6%])	Second line (n = 164 [32.4%])	
First diagnosis				
Age, median (IQR), y	52.4 (44.6-62.8)	52.6 (44.4-63.0)	52.2 (44.7-62.6)	.74
De novo metastatic	136 (26.9)	95 (27.8)	41 (25.0)	.58
Histotype				
IDC	373 (73.7)	261 (76.3)	112 (68.7)	.09
ILC	89 (17.6)	59 (17.3)	30 (18.4)	
Other	15 (3.0)	8 (2.3)	7 (4.3)	
Not available	29 (5.7)	14 (4.1)	15 (9.2)	
Disease-free interval after surgery, median (IQR), mo	71.9 (36.9-127.7)	66.4 (33.7-123.8)	81.0 (45.9-132.6)	.11
Beginning of CDK4/6i treatment				
Age, median (IQR), y	61.2 (52.1-69.4)	60.6 (50.9-69.9)	63.0 (55.2-71.7)	.009
Postmenopausal status	397 (78.5)	263 (76.9)	134 (81.7)	.40
ECOG PS				
0	408 (80.6)	289 (84.5)	119 (72.6)	.01
1	82 (16.1)	45 (13.2)	37 (22.6)	
≥2	11 (2.2)	5 (1.5)	6 (3.7)	
Not available	5 (1.0)	3 (0.9)	2 (1.2)	
Visceral involvement	267 (52.8)	173 (50.6)	94 (57.3)	.30
Type of CDK4/6i				
Palbociclib	339 (67.2)	208 (60.8)	131 (79.9%)	<.001
Ribociclib	117 (23.1)	96 (28.1)	21 (12.8)	
Abemaciclib	49 (9.7)	37 (10.8)	12 (7.3)	
Not available/unknown	1 (0.2)	1 (0.3)	0 (0.0)	
CDK4/6i dose reduction	185 (36.6)	125 (36.5)	60 (36.6)	0.49
Duration of CDK4/6i, median (IQR), mo	12.2 (5.9-21.1)	13.4 (7.0-21.3)	8.8 (4.4-20.4)	.006
Beginning of first treatment post CDK4/6i				
Age, median (IQR), y	62.4 (52.9-71.4)	61.7 (52.1-70.6)	63.4 (56.0-73.3)	.01
Visceral involvement	337 (66.6)	220 (64.3)	117 (71.3)	.14
No. of metastatic sites				
1	166 (32.8)	116 (33.9)	50 (30.5)	.02
2	167 (33.0)	100 (29.2)	67 (40.9)	
≥3	172 (34.0)	126 (36.8)	46 (28.0)	
Not available	1 (0.2)	0 (0.0)	1 (0.6)	
Type of treatment post CDK4/6i				
ET-based therapy	221 (43.7)	159 (46.5)	62 (37.8)	.08
CT-based therapy	285 (56.3)	183 (53.5)	102 (62.2)	

Abbreviations: CDKi, cyclin-dependent kinase inhibitor; CT, chemotherapy; ECOG PS, Eastern Cooperative Oncology Group Performance Status; ET, endocrine therapy; IDC, invasive ductal carcinoma; ILC, invasive lobular carcinoma.

At the time of post-CDK4/6i treatment initiation, median age was 62.4 (IQR, 52.9-71.4) years. Most patients (66.6%) exhibited visceral involvement. Moreover, 34.0% of the patients had 3 or more metastatic sites, which was more frequently observed in those who received first-line CDK4/6i (36.8%).

Following progression during ET plus CDK4/6i therapy, 221 patients (43.7%) received ET-based therapy and 285 patients (56.3%) received CT-based therapy. The ET-based therapy group included 114 patients (51%) who received everolimus plus exemestane and 100 patients (45%) who received ET alone (selective estrogen receptor degraders, aromatase inhibitors, or megestrol acetate). The CT-based group included 106 patients receiving single-agent or combination intravenous CT and 179 patients who received oral CT (capecitabine or other drugs, alone or in combination). Seven patients were excluded from the therapy-based PFS and OS analyses due to treatment fragmentation (only 6 patients treated with fulvestrant plus alpelisib and 1 patient receiving letrozole plus alpelisib), but they were included in all other survival analyses (Table 1). Missing data for every variable are reported in Table 1 and the rate of loss to follow-up was 3% (n = 15) for OS.

### Clinical Outcomes

Table 2 and Table 3 report the results of multivariable models, with estimated HRs for PFS (median follow-up, 4.75 [IQR, 2.82-9.31] months) and OS (median follow-up, 14 [IQR, 7.77-23.93] months) adjusted for the duration of prior ET plus CDK4/6i agents, type of therapy administered after progression to ET plus CDK4/6i, and demographic and clinicopathologic characteristics. The median PFS in the overall population was 5.44 months (95% CI, 4.59-5.97). In particular, the median PFS of patients treated with oral CT was 6.89 (95% CI, 5.31-8-82) months compared with 5.44 (95% CI, 3.90-6.16) months in the group of patients treated with intravenous CT, 4.82 months (95% CI, 4.09-6.66) in those who received everolimus plus exemestane, and 3.87 months (95% CI, 3.44-4.69) in patients treated with mono-endocrine therapy.

**Table 2.** Multivariable Cox Proportional Hazards Model Evaluating PFS and OS in Post-CDK4/6i Treatment

Variable	PFS		OS	
	HR (95% CI)	*P* value	HR (95% CI)	*P* value
Age	0.99 (0.98-1.00)	.03	1.01 (0.99-1.02)	.22
De novo MBC				
No	1 [Reference]	NA	1 [Reference]	NA
Yes	1.25 (1.01-1.54)	.04	1.25 (0.93-1.68)	.14
CDK4/6i duration, mo				
<12	1 [Reference]	NA	1 [Reference]	NA
≥12	0.89 (0.73-1.08)	.24	0.55 (0.41-0.73)	<.001
Visceral metastases				
No	1 [Reference]	NA	1 [Reference]	NA
Yes	1.45 (1.17-1.80)	.008	1.63 (1.19-2.24)	.002
Type of treatment				
Oral CT	1 [Reference]	NA	1 [Reference]	NA
Intravenous CT	1.45 (1.11-1.89)	.006	1.35 (0.95-1-92)	.09
Everolimus plus exemestane	1.38 (1.06-1.78)	.02	0.80 (0.54-1.17)	.24
ET only	1.38 (1.05-1.89)	.02	0.99 (0.68-1.45)	.95

Abbreviations, CDK4/6i, cyclin-dependent kinase inhibitor; CT, chemotherapy; ET, endocrine therapy; HR, hazard ratio; MBC, metastatic breast cancer; NA, not applicable; OS, overall survival; PFS, progression-free survival.

**Table 3.** Multivariable Cox Proportional Hazards Model Evaluating PFS and OS in Post-CDK4/6i Treatment According to Visceral Involvement

Variable	PFS		OS	
	HR (95% CI)	*P* value	HR (95% CI)	*P* value
Patients with visceral metastases (n = 337)				
Age	0.99 (0.98-0.99)	.04	1.01 (0.99-1.02)	.19
De novo MBC				
No	1 [Reference]	NA	1 [Reference]	NA
Yes	1.26 (0.97-1.64)	.08	1.14 (0.80-1.62)	.47
CDK4/6i duration				
<12 mo	1 [Reference]	.51	1 [Reference]	.002
≥12 mo	0.92 (0.72-1.18)		0.59 (0.42-0.82)	
Type of treatment				
Oral CT	1 [Reference]	NA	1 [Reference]	NA
Intravenous CT	1.57 (1.16-2.13)	.004	1.52 (1.03-2.24)	.04
Everolimus plus exemestane	1.63 (1.18-2.26)	.003	0.96 (0.61-1.49)	.85
ET only	1.67 (1.18-2.34)	.004	1.07 (0.67-1.71)	.79
Patients without visceral metastases (n = 169)				
Age	0.99 (0.98-1.01)	.28	1.00 (0.98-1.02)	.88
De novo MBC				
No	1 [Reference]	NA	1 [Reference]	NA
Yes	1.32 (0.90-1.94)	.15	1.55 (0.87-2.77)	.14
CDK4/6i duration, mo				
<12	1 [Reference]	.28	1 [Reference]	.008
≥12	0.83 (0.58-1.17)		0.45 (0.25-0.81)	
Type of treatment				
Oral CT	1 [Reference]	NA	1 [Reference]	NA
Intravenous CT	1.52 (0.80-2.90)	.20	0.99 (0.34-2.88)	.99
Everolimus plus exemestane	1.08 (0.70-1.78)	.72	0.53 (0.23-1.20)	.13
ET only	1.02 (0.66-1.59)	.93	0.78 (0.40-1.52)	.47

Abbreviations, CDK4/6i, cyclin-dependent kinase inhibitor, CT, chemotherapy, ET, endocrine therapy, HR, hazard ratio; MBC, metastatic breast cancer; NA, not applicable; OS, overall survival; PFS, progression-free survival.

### Multivariable Analysis for PFS in the Overall Population

**Figure 1.**
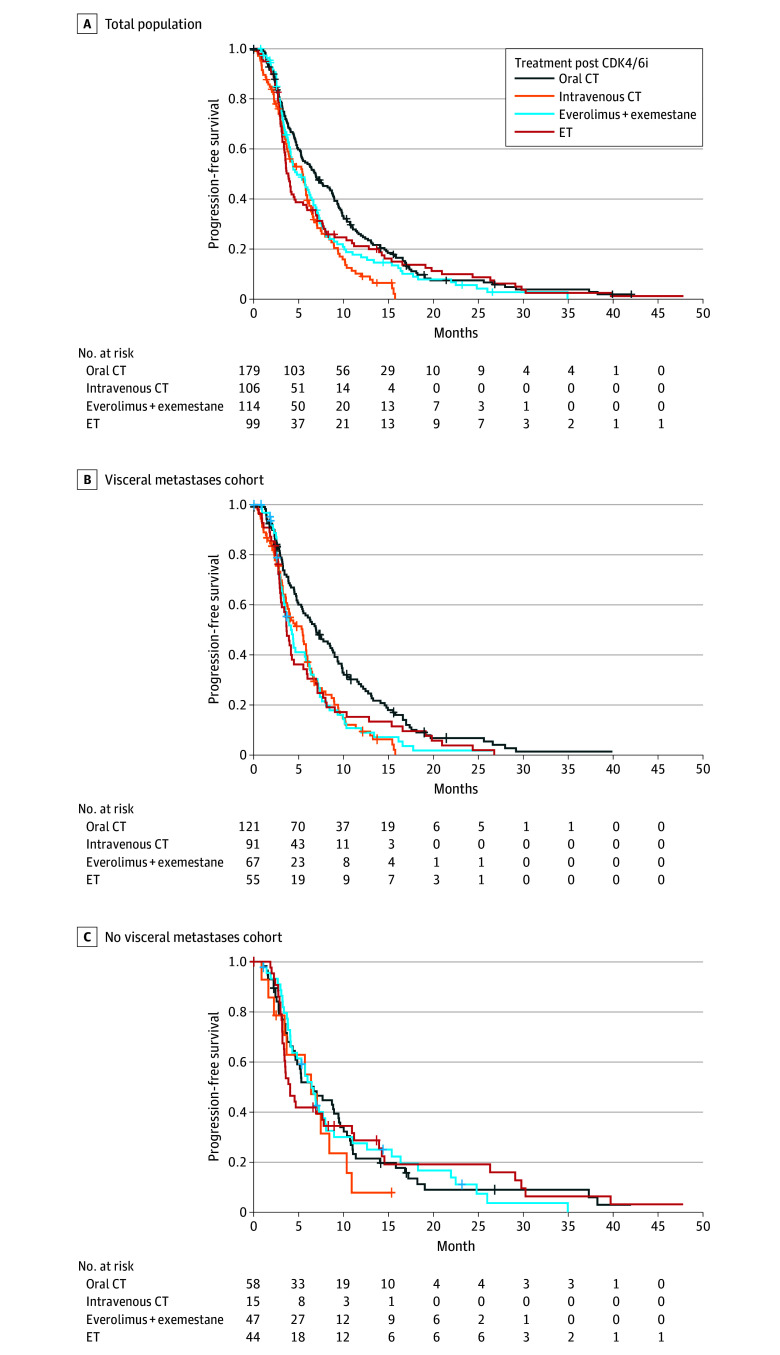
Progression-Free Survival After Cyclin-Dependent Kinase Inhibitor (CDK4/6i) According to Treatment Type A, Progression-free survival in the overall population. Median progression-free survival: oral chemotherapy (CT) 6.89 (95% CI, 5.31-8.82) months; intravenous CT (alone or in combination), 5.44 (95% CI, 3.90-6.16) months; everolimus plus exemestane, 4.82 (95% CI, 4.09-6.66) months; endocrine therapy (ET), 3.87 (95% CI, 3.44-4.69) months; *P* = .005. B, Patients with visceral metastases. Median progression-free survival: oral CT, 6.91 (95% CI, 5.44-8.98) months; intravenous CT (alone or in combination), 5.40 (95% CI, 3.77-5.97) months; everolimus plus exemestane, 4.13 (95% CI, 3.34-6.23) months; ET, 3.63 (95% CI, 3.02-5.51) months; *P* < .001. C, Patients with no visceral metastases. Median progression-free survival: oral CT, 6.69 (95% CI, 4.66-9.64) months; intravenous CT (alone or in combination), 6.39 (95% CI, 3.67-not achieved) months; everolimus plus exemestane, 6.46 (95% CI, 4.82-8.95) months; ET, 4.07 (95% CI, 3.41-10.95) months; *P* = 80. Global *P* value adjusted for multiple pairwise comparisons using the Holm correction.

In the multivariable model, de novo metastatic disease (HR, 1.25; 95% CI, 1.01-1.54; *P* = .04) and the presence of visceral involvement (HR, 1.45; 95% CI, 1.17-1.80; *P* = .008) were independently associated with a higher risk of disease progression or death (Table 2). In addition, advanced age was associated with better PFS (HR, 0.99; 95% CI, 0.98-1.00; *P* = .03). Compared with oral CT, the use of intravenous CT (HR, 1.45; 95% CI, 1.11-1.89; *P* = .006), everolimus plus exemestane (HR, 1.38; 95% CI, 1.06-1.78; *P* = .02), or single-agent ET (HR, 1.38; 95% CI, 1.05-1.89; *P* = .02) was associated with a higher risk of disease progression and a lower median PFS both for endocrine-based and intravenous CT-based therapy (Table 2, Figure 1A).

### Multivariable Analysis for PFS According to Visceral Involvement

In patients with visceral involvement, older patient age was associated with better PFS (HR, 0.99; 95% CI, 0.98-0.99; *P* = .04). Patients treated with oral CT were at a lower risk of disease progression or death compared with patients who received intravenous CT (HR, 1.57; 95% CI, 1.16-2.13; *P* = .004), everolimus plus exemestane (HR, 1.63; 95% CI, 1.18-2.26; *P* = .003), or ET only (HR, 1.67; 95% CI, 1.18-2.34; *P* = .004) (Table 3). This also reflects the better median PFS in the cohort of patients with visceral metastases, as shown in Figure 1B. Conversely, in the 169 patients without visceral involvement, these treatments showed similar outcomes (Table 3, Figure 1C).

### Multivariable Analysis for OS

**Figure 2.**
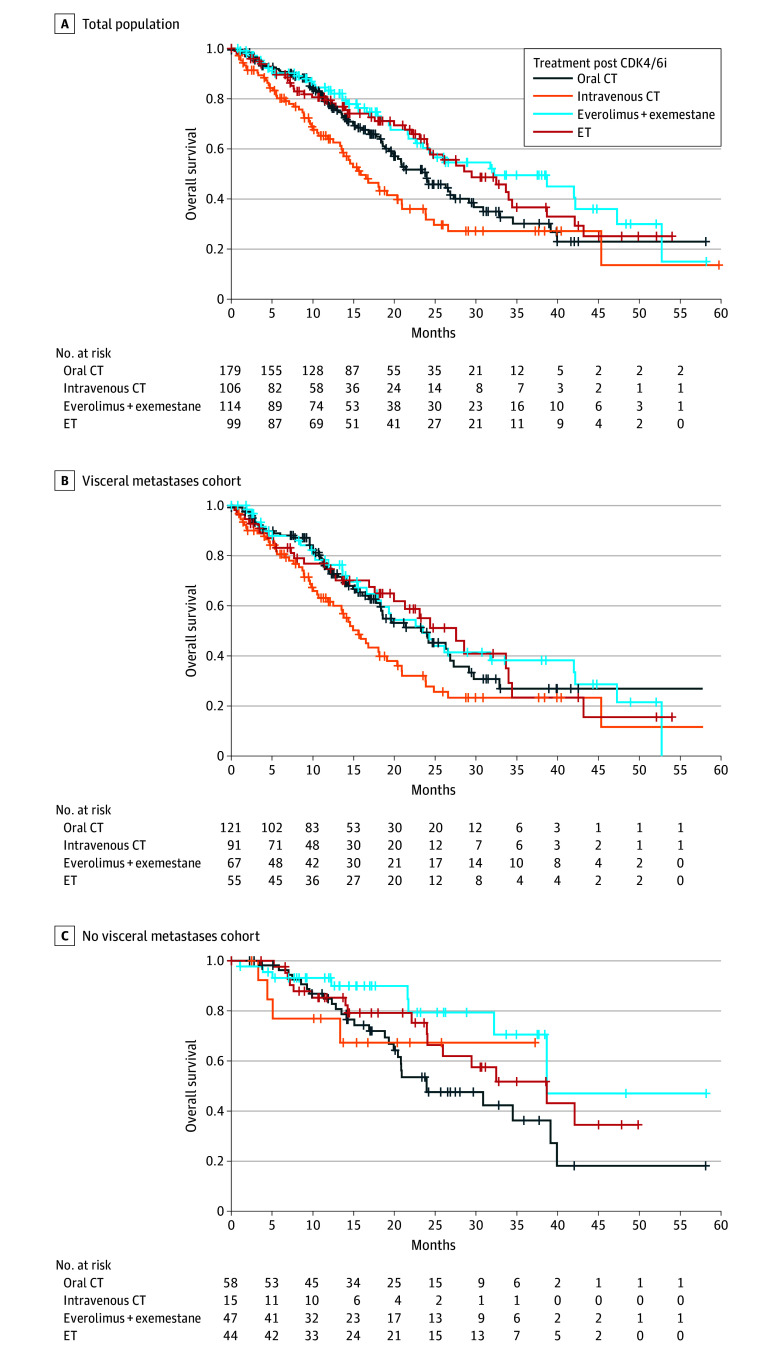
Overall Survival After Cyclin-Dependent Kinase Inhibitor (CDK4/6i) According to Treatment Type A, Overall survival in the total population. Median overall survival: oral chemotherapy (CT), 23.93 (95% CI, 19.67-27.24) months; intravenous CT (alone or in combination), 15.87 (95% CI, 13.51-20.88) months; everolimus plus exemestane, 32.20 (95% CI, 22.59-42.25) months; endocrine therapy (ET), 29.44 (95% CI, 23.97-38.66) months; *P* < .001. B, Patients with visceral metastases. Median overall survival: oral CT, 23.38 (95% CI, 18.23-27.25) months; intravenous CT (alone or in combination), 15.57 (95% CI, 11.84-19.08) months; everolimus plus exemestane, 23.48 (95% CI, 16.59-41.97) months; ET, 27.54 (95% CI, 15.57-33.97) months; *P* = .05. C, Patients without visceral metastases. Median overall survival: oral CT, 23.93 (95% CI, 19.90-39.11) months; intravenous CT (alone or in combination), not achieved (NA) (95% CI, 5.08-NA) months; everolimus plus exemestane, 38.66 (95% CI, 24.03-NA) months; ET, 38.66 (95% CI, 32.20-NA) months; *P* = .09. Global *P* value adjusted for multiple pairwise comparisons using the Holm correction.

A duration of prior ET plus CDK4/6i treatment of at least 12 months was independently associated with a lower risk of death (HR, 0.55; 95% CI, 0.41-0.73; *P* < .001), whereas the presence of visceral metastases was associated with worse OS compared with the absence of visceral involvement (HR, 1.63; 95% CI, 1.19-2.24; *P* = .002) (Table 2). In our analysis there was no significant difference in survival according to post-CDK4/6i treatment (Table 2, Figure 2A).

### Multivariable Analysis for OS According to Visceral Involvement

Longer duration of prior ET plus CDK4/6i treatment was associated with a lower risk of death both in patients with visceral metastases (HR, 0.59; 95% CI, 0.42-0.82; *P* = .002) and in patients without visceral involvement (HR, 0.45; 95% CI, 0.25-0.81; *P* = .008) (Table 3**)**. In addition, intravenous CT showed an independent association with worse OS compared with oral CT only in patients with visceral metastases (HR, 1.52; 95% CI, 1.03-2.24; *P* = .04).

### Clinical Outcomes in Patients With Known Germinal *BRCA1/2* Status

In our dataset, the germinal status of *BRCA1/2* genes was available for 103 patients. Of these, 16 patients (15%) were carriers of *BRCA1* or *BRCA2* pathogenic or likely pathogenic variants, while 87 (85%) had wild-type *BRCA1/2* genes. The *BRCA1/2* carriers had a higher likelihood of having received a diagnosis of de novo metastatic disease compared with patients with tumor relapsing after local therapies (43.8% vs 18.4%; *P* = .045).

At the beginning of the systemic therapy after tumor progression during ET plus CDK4/6i treatment, patient age, the number of metastatic sites, the presence of visceral involvement, and the type of treatment (ET vs CT based) were similarly distributed in *BRCA1/2* mutated vs wild-type groups. Patients who carried a *BRCA1/2* pathogenic variant had a statistically significant shorter median PFS (3.95 months; 95% CI, 3.25-5.31 months) compared with patients with wild-type *BRCA1/2* (5.97 months; 95% CI, 4.10-6.92 months) (*P* = .03). *BRCA1/2* pathogenic variant carriers also had shorter median OS (26.9 months; 95% CI, 23.1-not achieved) compared with *BRCA1/2* pathogenic variant noncarriers (OS not achieved; 95% CI, 30.8-not achieved) (*P* = .34).

## Discussion

The optimal treatment sequence following progression during ET plus CDK4/6i treatment in patients with hormone receptor–positive/*ERBB2*–negative metastatic breast cancer remains a matter of debate. The primary objective of this cohort study in the clinical practice setting was to investigate the potential association of clinicopathologic characteristics and treatment type with survival outcomes in a large dataset of patients with hormone receptor–positive, *ERBB2*–negative metastatic breast cancer undergoing disease progression with ET plus CDK4/6i treatment, with the goal of providing useful insights to support the decision-making process in this challenging clinical practice setting.

Approximately two-thirds of the patients included in this study received palbociclib as CDK4/6i treatment in combination with ET. This can be attributed to the fact that the study included patients with metastatic breast cancer diagnosed between 2015 and 2023, and palbociclib was the first CDK4/6i targeting agent to be authorized by the European Regulatory Agency in 2016.^33^ The median duration of treatment with CDK4/6i agents was 13.4 (IQR, 7.0-21.3) months for patients who received first-line ET plus CDK4/6i and 8.8 months (IQR, 4.4-20.4) in patients treated with second-line ET plus CDK4/6i. The duration of ET plus CDK4/6i treatment was slightly lower than that reported in clinical trials, and this may be attributable to the fact that we excluded patients who did not experience disease progression and were still deriving benefit from the drug at the time of data collection.^6,7,8,11,13,34,35,36^ Median PFS after tumor progression during ET plus CDK4/6i treatment was 5.44 months, which is in line with previous data ranging from 4 to 8 months.^34,35,36,37^ For OS, we found that patients who had received CDK4/6i agents for at least 12 months exhibited longer OS than those who had received CDK4/6i agents for less than 12 months at both univariate and multivariable analyses. Longer tumor control during ET plus CDK4/6i therapy may reflect a more indolent and endocrine-sensitive disease, which could imply a better response to subsequent treatments and longer disease control. Consistent with this interpretation, a subgroup analysis of the EMERALD trial showed that patients with hormone receptor–positive, *ERBB2*–negative metastatic breast cancer and *ESR1* mutations who had continued ET plus CDK4/6i treatment for 12 months or more achieved significantly higher benefit from the oral selective estrogen receptor degrader elacestrant.^37^ By contrast, a subgroup analysis from the BYLieve trial found no association between the duration of prior ET plus CDK4/6i therapy and the efficacy of ET plus alpelisib in patients with *PIK3CA*-mutated hormone receptor–positive, *ERBB2*–negative metastatic breast cancer.^38^ These conflicting results suggest that the duration of ET plus CDK4/6i treatment may be differentially associated with the efficacy of diverse antitumor agents. Nevertheless, the influence of the duration of CDK4/6i exposure on OS in patients with hormone receptor–positive, *ERBB2*–negative metastatic breast cancer remains unclear and needs further investigation.

In our study, patients with de novo metastatic breast cancer exhibited a slightly yet statistically significant lower PFS compared with patients with relapsed tumors. This was observed only in premenopausal patients. This finding is in contrast with previous reports, which typically suggest better outcomes for de novo metastatic breast cancer.^39,40^ One possible explanation for this discrepancy is that younger patients are more likely to carry *BRCA1/2* pathogenic variants,^41^ which are associated with an aggressive disease course^42^ and possible diminished response to both ET and CDK4/6i treatment.^43,44,45^

Two-thirds of patients included in our study exhibited visceral metastases, with one-third of them having at least 3 metastatic sites. Several studies have reported that the presence of visceral metastases is associated with shorter survival compared with nonvisceral metastases in patients with breast cancer.^46,47,48,49^ Our study supports the negative association of visceral involvement with both PFS and OS in patients progressing during ET plus CDK4/6i treatment. The presence of visceral metastases typically reflects more aggressive tumor biologic subtypes, and its treatment remains an unmet need for patients with metastatic breast cancer.^26,50^ Although our study excluded patients who received a subsequent CDK4/6i after progressing during a first CDK4/6i, the potential use of sequential CDK4/6i therapy warrants mention.^51^ As found in the postMONARCH trial, abemaciclib combined with fulvestrant after progression during ET plus CDK4/6i treatment (primarily palbociclib, in a setting comparable to our study) showed a PFS benefit over fulvestrant alone across major subgroups, including patients with *ESR1* or *PIK3CA* mutations (data not collected in our study). In patients with visceral metastases, the median PFS in the combination arm was 5.4 (95% CI, 3.7-5.9) months compared with 3.7 (95% CI, 2.0-5.4) months in the fulvestrant-alone arm (HR, 0.87; 95% CI, 0.64-1.17).^25^ In this clinical practice setting, our study found that oral CT resulted in a better PFS compared with intravenous CT-based or standard ET-based treatments. Most patients in the oral CT group were treated with capecitabine, either alone or in combination with oral vinorelbine and cyclophosphamide, achieving a median PFS of 6.89 months. This outcome is comparable to the PFS reported for capecitabine in the DESTINY Breast-06 trial, which investigated trastuzumab deruxtecan vs the investigator’s choice of chemotherapy in pretreated patients with hormone receptor–positive, *ERBB2* low and ultralow metastatic breast cancer.^29^ However, this PFS benefit did not translate into a similar benefit for OS, suggesting that the choice between ET and oral CT after tumor progression during ET plus CDK4/6i treatment did not significantly influence the overall course of metastatic disease in the broader population. This discrepancy may be partially explained by the smaller number of OS events compared with PFS events, indicating the need for a longer follow-up to achieve OS data maturity. Conversely, intravenous CT was associated with significantly worse OS in patients with visceral metastases compared with oral CT. While intravenous and oral CT have comparable antitumor efficacy, oral CT offers advantages in terms of convenience, ease of administration, fewer adverse effects, and better quality of life.^52^ Generally, clinicians reserve monotherapy or polytherapy intravenous CT regimens for patients with a high visceral disease burden or rapidly progressing disease, which may explain the shorter OS observed in our study, likely influenced by patient selection bias. In the DESTINY Breast-06 trial, trastuzumab deruxtecan demonstrated a significant and clinically meaningful PFS benefit over CT (oral or intravenous) in patients with hormone receptor–positive, *ERBB2* low and ultralow metastatic breast cancer after progression to at least 2 lines of ET or 1 line with early progression. Although our study did not include patients receiving trastuzumab deruxtecan after progression during CDK4/6i treatment, trastuzumab deruxtecan could be considered the new standard of care in this setting for patients with and without visceral metastases.^29,53^

Among the patients in our study, 16 were carriers of a *BRCA1/2* pathogenic variant. These patients had demographic and clinicopathologic characteristics similar to those with wild-type *BRCA1/2*, including age, menopausal status, ECOG performance status, visceral involvement, and duration of CDK4/6i therapy. However, they exhibited lower median PFS, which may reflect the intrinsic aggressiveness of *BRCA1/2*-mutated breast cancer.^34,54^ Additionally, none of these patients received a poly (ADP-ribose) polymerase inhibitor and only 2 received carboplatin-based CT as post-CDK4/6i treatment.^35,55^ These findings should be interpreted with caution due to the small number of *BRCA1/2* pathogenic variant carriers (n = 16) and the variability in treatment received.

### Limitations

This study has several limitations. First, the retrospective design introduces the possibility of biases, including selection bias, and potential uncontrolled confounding factors. We sought to mitigate these limitations by using multivariable and subgroup analyses. Additionally, patients who received a second CDK4/6i after progression during a first CDK4/6i treatment were excluded. The retrospective design and the treatment period also precluded the collection of data on relevant tumor genomic alterations, such as *PIK3CA*, *AKT*, *PTEN*, and *ESR1*, which limited our ability to assess the potential association between these tumor mutations and treatment outcomes. Despite these limitations, to our knowledge, this is one of the largest series in the post-CDK4/6i setting, offering valuable insights into factors involved in treatment efficacy that can help benchmark and guide clinical decision-making.

## Conclusions

To our knowledge, this study represents one of the largest analyses in the clinical practice setting of post-CDK4/6i therapeutic outcomes in hormone receptor–positive, *ERBB2*–negative metastatic breast cancer, providing critical insights that may shape future treatment strategies. In this cohort study of 506 patients diagnosed with hormone receptor–positive, *ERBB2*–negative metastatic breast cancer progressing during ET plus CDK4/6i treatment, our findings suggest that oral CT could be a preferred option for select patients with visceral metastases, offering comparable survival outcomes with potentially fewer adverse effects and greater convenience. However, these results may be influenced by selection bias and should be interpreted with caution, requiring case-by-case discussions to identify the most appropriate individualized treatment. Further research is warranted to confirm these findings and explore the potential benefits of personalized treatment approaches, particularly considering the duration of CDK4/6i therapy and the presence of visceral metastases.

## Notes

Supplement.
Data Sharing Statement
