# Supplementary material for: Survival Following CDK4/6 Inhibitor Therapy for Hormone Receptor–Positive, ERBB2–Negative Metastatic Breast Cancer
Source: JAMA Netw Open. 2025 Feb 21;8(2):e2461067. doi: 10.1001/jamanetworkopen.2024.61067 (PMC11846014; doi:10.1001/jamanetworkopen.2024.61067)
Supplement: Supplement. — Data Sharing Statement [file jamanetwopen-e2461067-s001.pdf]

Data Sharing Statement

Berton Giachetti. Survival Following CDK4/6 Inhibitor Therapy for Hormone Receptor–Positive, ERBB2–Negative Metastatic Breast Cancer. *JAMA Netw Open*. Published February 21, 2025. doi:10.1001/jamanetworkopen.2024.61067

Data
Data available: No
